# Legal and Forensic Issues in Telepsychiatry

**DOI:** 10.1192/j.eurpsy.2022.185

**Published:** 2022-09-01

**Authors:** K. Goethals

**Affiliations:** Antwerp University Hospital, University Forensic Centre, Edegem, Belgium

**Keywords:** Prisoners, telepsychiatry, courts, confidentiality

## Abstract

Covid-19 has induced many changes to society, including some in the practice of medicine and psychiatry. Among them is increasing use of telecommunications. A previous editorial outlined the possible uses and dangers of telemedicine with prisoners (Gunn et al 2020). Forensic psychiatry is also concerned with providing expert evidence to courts and other arbitration bodies and, increasingly, these bodies too are relying on such technology. Further in addition to traditional paper-style records (many now held electronically rather than literally on paper) there is increasing use of video recording of interviews, of day to day behaviour on secure hospital units and by bodycams when intervening in a tense, potentially violent situation. To what extent are these being used in court? Is there a European framework for guiding us on how to proceed? How has this been interpreted to date in countries across Europe? In this paper these issues will be addressed.

**Disclosure:**

No significant relationships.

